# President’s Address

**Published:** 1904-10-15

**Authors:** Charles C. Noble

**Affiliations:** Detroit


THE
### DENTAL REGISTER.

Vol. LVIII. October 15, 1904. No. 10.

### PRESIDENT'S ADDRESS.

BY CHARLES C. NOBLE, D. D. S. DETROIT.

Read before the Michigan Dental Association, June 28, 1904.

MEMBERS OF THE MICHIGAN DENTAL ASSOCIATION:—We have come together for the forty-eighth annual meeting of this Association and to discuss methods for the advancement of our profession, also that we may, by free interchange of ideas, perfect ourselves in such special lines of practice that we may be of more benefit to those who intrust themselves to our ministrations.

Our Association may be reported to be in a flourishing condition, but the membership is not what it should be in view of the registered practitioners in the State. This matter has been presented to you several times, but I consider it of so much importance that I take the liberty of again bringing it before you, and urge some action by this meeting which will materially increase our regular membership as well as attendance.

The tendency at the present time in our profession seems to be to specialize in some particular branch of the profession with the idea of rendering a higher grade of skilled service. The field of dentistry offers great opportunity for advancement in general practice, and as the great majority of the profession are doing a general practice, there is need for meetings of this character if we wish to keep up to the times, to say nothing of making desirable advancement.

By exchange of thought in papers and discussions, and witnessing clinics and exhibits, we are all spurred to greater effort, and thus we may make our work professional in every sense of the word. We have, throughout the State, numerous local societies that are doing good work, and I believe that through these societies this Association can be strengthened. I would suggest that a committee, consisting of the President and Secretary of this Association, and two members from each local society in the State, be appointed to take up this matter of membership, and that the work be pushed as vigorously as possible, this committee to report at the next annual meeting.

The dental meetings in the several States have become of such magnitude and importance that I think the time has now arrived when much benefit will be derived by having some arrangement made, especially with adjoining States, whereby the meetings may not conflict with each other as to time of sessions. If a so-called circuit of meetings could be arranged, it would give the members a chance to attend the different meetings and we should be able to provide clinicians more easily, and to get more and better exhibits, as the expense of traveling would be materially reduced. This matter can easily be arranged by the secretaries of the different Associations, and I offer it for your consideration and future action.

The State Board of Examiners in dentistry has, in the past, been severely criticized, and some matters pertaining to their acts have been referred to your Board of Directors for investigation. This has been done, and we would report that we believe the present Board is worthy of the hearty support and co-operation of this Association. We should work together for the mutual improvement and betterment of the practice of our profession generally, and as the State Board is also formed for this latter purpose we should work hand in hand and endeavor to give the people of Michigan the standard of proficency in practice their law demands.

One of the most important meetings in the history of dentistry will be the International Congress held at St. Louis the latter part of August. The dental profession of the United States has a reputation to sustain that should make every one of us put forward his best efforts to make this meeting a grand success. Each and every one should make it a personal matter to give all moral and financial support possible, and this Association, as a whole, should give such financial aid as is deemed consistent.

The Tri-State meetings of Michigan, Ohio and Indiana, instituted by Michigan nine years ago, proved such successful gatherings that is it deemed wise to continue them, as they are eagerly expected, not only by the members of these three State Associations but by many others as well. Action should be taken at this meeting looking toward the formation of committees in the different States to prepare programs etc., for the meeting next year. The previous meetings have been such grand successes that it will entail lots of hard work on the part of every member of this Association if we shall sustain our reputation for hospitality and excellence of program made by the first Tri-State meeting.

The program presented for your consideration at this meeting is one that I am sure will be of great benefit and interest, and the gentlemen who prepared it are entitled to much credit for their earnest and pains-taking effort.

I thank you for the honor you have conferred upon me, and trust you will unite with me in making this meeting one of the best ever held by the Michigan Dental Association.

In compliance with the recommendations of the President's address one hundred dollars was appropriated to the expenses of the International Dental Congress.

The following committee was appointed to serve as a general committee of organization for the Tri-State meeting to be held next year: Drs. C. C. Noble, H. C. Raymond, C. H. Worboys, J. J. Green, A. F. Monroe.

The suggestion to make an effort to increase the membership of the society was also adopted and will be worked up by the Board of Directors through the President and Secretary as a special committee.—EDITOR.

